# The Emerging Roles of Pellino Family in Pattern Recognition Receptor Signaling

**DOI:** 10.3389/fimmu.2022.728794

**Published:** 2022-02-07

**Authors:** E Zhang, Xia Li

**Affiliations:** ^1^ Marine College, Shandong University, Weihai, China; ^2^ School of Pharmaceutical Sciences, Shandong University, Jinan, China

**Keywords:** Pellino1, Pellino2, Pellino3, TLR, NLR, RIP, PRRs

## Abstract

The Pellino family is a novel and well-conserved E3 ubiquitin ligase family and consists of Pellino1, Pellino2, and Pellino3. Each family member exhibits a highly conserved structure providing ubiquitin ligase activity without abrogating cell and structure-specific function. In this review, we mainly summarized the crucial roles of the Pellino family in pattern recognition receptor-related signaling pathways: IL-1R signaling, Toll-like signaling, NOD-like signaling, T-cell and B-cell signaling, and cell death-related TNFR signaling. We also summarized the current information of the Pellino family in tumorigenesis, microRNAs, and other phenotypes. Finally, we discussed the outstanding questions of the Pellino family in immunity.

## Introduction

Immune responses are mainly divided into innate immunity and acquired immunity. Innate immunity can respond rapidly to pathogens as the first line of defense mediated by macrophages, dendritic cells, neutrophils, epithelial, and endothelial cells. It utilizes germ-line encoded pattern recognition receptors (PRRs) to detect conserved microbial components known as pathogen-associated molecular patterns (PAMPs) or endogenous ‘alarmins’ released during infection and inflammation. Toll-like receptors (TLRs), nucleotide-binding oligomerization domain-like receptors (NLRs), retinoic acid-inducible gene I-like receptors (RLRs), C-type lectin receptors (CLRs), DNA sensors, and melanoma 2-like receptors are not part of the PRRs (AIM-2-like receptors). Mammalian TLRs recognize bacteria and nucleic acids and sense inflammation caused by bacteria through binding to ligands on the cell surface and in the nuclear body. NLRs and RLRs, on the other hand, detect nucleic acids in the cytosol (1–3). PRRs mediate their biological functions by activating transcription factors such as nuclear factor−κB (NF-κB), activator protein-1 (AP-1), and interferon-regulatory factors (IRFs) to drive proinflammatory and interferon (IFN) gene expression (1–4).

Ubiquitination, a posttranslational modification involving the conjugation of the 76 amino acid proteins to the lysine residue of other proteins, is catalyzed by the sequential action of ubiquitin-activating (E1), ubiquitin-conjugating (E2), and ubiquitin-ligating (E3) enzymes. Ubiquitin contains seven lysine residues and one N-terminal methionine (M1) residue, each of which can be attached to another ubiquitin moiety. The presence of these lysine residues and the M1 forms a variety of ubiquitin chains (K6-, K11-, K27-, K29-, K33-, K48-, K63-, M1-linked ubiquitin chains and mixed ubiquitin chains), which are recognized by substrate proteins with linkage-specific ubiquitin-binding domains to trigger multiple biological functions such as K48- and K11-linked chains for protein degradation, M1 or K63-linked chains for signal transduction (5). The substrate specificity of ubiquitination is mainly determined by E3s, which directly catalyzes ubiquitin transfer from E2s to the substrates (6). In particular, the Pellino family, a novel E3 ubiquitin ligase family (7–9), has been implicated in the regulation pattern recognition receptors (PRRs) signaling pathway of immunity.

**Figure 1:**
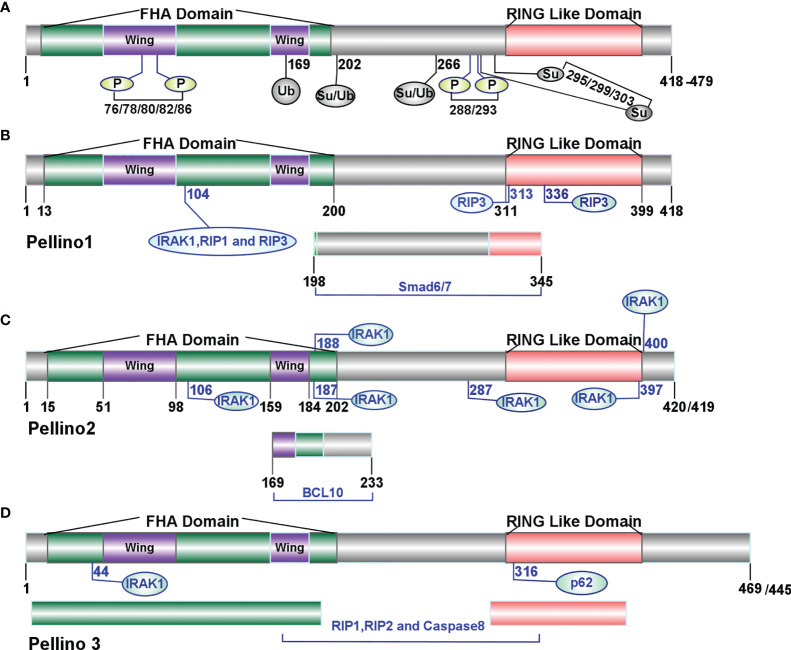
Molecular features of the Pellino family (9). **(A)** The structure of the Pellino family. In mammals, the Pellino family comprises three family members (Pellino1, Pellino2, and Pellino3) with an amino acid length ranging from 418 to 479. The Pellino family shows a highly similar primary structure with a C-terminal RING-like domain mediating K11, K48, and K63 linked conjugation of polyubiquitination and a cryptic phosphothreonine-binding N-terminal hidden split forkhead associated (FHA) domain attached by a “wing” or appendage structure. IRAK1 and IRAK4 can phosphorylate the Pellino family on Ser-76, Ser-78, Thr-80, Ser-82, and Thr-86. Individual site Ser-76, Thr-86, Thr-288, or Ser-293 or a combination of Ser-78, Thr-80, and Ser-82 is necessary to activate the Pellino family (17) fully. IKKϵ/TBK1 activates Pellino1 *in vitro* by phosphorylating Ser76, Thr288, and Ser293 (18). **(B)** The sites of Pellino1 interacting with other proteins. The amino acids length of Pellino1 is 418 in both humans and mice. The 104th site of the FHA domain and 313th/336th sites of the RING-like domain are crucial to K48-linked polyubiquitylation of IRAK1, RIP1, and RIP3 (19). The region between residues 198 and 345 is essential to the interaction between Pellino1 and Smad6/7 (20, 21). **(C)** The sites of Pellino2 interacting with other proteins. The amino acids length of Pellino2 is 420 in humans and 418 in mice. The points 106, 187, 188, 287, 397, and 400 are essential to the interaction between Pellino2 and IRAK1 (16, 22). The range between residues 169 and 233 is essential to the interaction between Pellino2 and BCL10 (23). **(D)** The sites of Pellino3 interacting with other proteins. The amino acid length of Pellino3 is 469 in humans and 445 in mice. Residue 44 is essential for the binding of Pellino3 to IRAK1 (24). Residue 316 is essential to Pellino3 autophagy-dependent degradation *via* p62 (25). The FHA and RING-like domains are responsible for the interaction between RIP1 and caspase-8 (26, 27).

Pellino (*Drosophila* Peli, Human PELI, Mouse *Peli*), first discovered in *Drosophila*, is a novel and evolutionarily conserved protein with 424 amino acid residues and an estimated molecular weight of 47 kDa (10). The Pellino family-related sequences are conserved in different species (11, 12). The identical sequence shared between *C. elegans* and *Drosophila* is 47%, and between *C. elegans* and Human is 40% (12). In mammals, the Pellino family has three sequence-conserved members, Pellino1 (13), Pellino2 (14), and Pellino3 (two splicing variants Pellino3a and Pellino3b) (15), located on chromosomes 2, 14, and 11 (13) respectively, with an amino acid length ranging from 418 to 479 (16). Mouse Pellino1 and Pellino2 possess 75% sequence similarity, whereas Pellino3 shares 84 and 85% similarity with Pellino1 and Pellino2, respectively (13). Each member of the Pellino family shows a highly similar primary structure with a C-terminal RING-like domain mediating K11, K48, and K63 linked conjugation of polyubiquitination (7) and a cryptic phosphothreonine-binding N-terminal hidden split forkhead associated (FHA) domain attached by a “wing” or appendage structure (16) (**Figure 1**). The “wing” can interact with phosphothreonine residues of proteins such as interleukin-1 receptor-associated kinase 1 (IRAK1) and interleukin-1 receptor-associated kinase 4 (IRAK4), which in turn phosphorylate Pellino1, Pellino2, and Pellino3 (9, 16, 24, 28–31).

The Pellino family was thought to be a kind of “scaffolding” protein in the signaling process of Toll-like receptors and interleukin-1 receptors (TLR/IL-1R) (15) by interacting with multiple intermediates such as IRAK4, IRAK1, TGF-beta activated kinase 1 (TAK1), TAK1 binding protein 1 (TBK1), receptor-interacting protein kinase (RIPK or RIP) and TNF receptor-associated factor 6 (TRAF6) (15, 32–38). Subsequent research showed that the Pellino family acted as a novel interesting new gene (RING) E3 ubiquitin ligases (7, 14, 39) rather than scaffold proteins (15). Similar to classical C3HC4 RING structure, the carboxyl termini of the Pellino family possesses two stable Cys-Gly-His motifs and two conserved Cys-Pro-X-Cys motifs, which determine and characterize the feature of the RING class of E3 ligase (12). The Pellino family exerts their E3 ubiquitin ligase activity through its phosphorylation form. Some proteins can phosphorylate the Pellino family, such as IRAKs (IRAK1 and IRAK4), TAK1, TBK1, and IκB kinase ϵ (IKKϵ) (15, 32–36). Upon stimulation by interleukin-1(IL-1), tumor necrosis factor α(TNFα), lipopolysaccharide (LPS) or polyinosinic–polycytidylic acid [poly (I:C)] (18, 35), Pellino1 can be fully activated by phosphorylation at some different sites (Ser-76, Thr-86, Thr-288, or Ser-293) or a combination of other sites (Ser-78, Thr-80, and Ser-82) (18, 31). As a critical family of E3 ubiquitin ligases, the Pellino family can mediate K11, K48, and K63 linked polyubiquitination (7). Pellino1 can combine with E2 conjugating complex ubiquitin-conjugating enzyme 13 (Ubc13)–ubiquitin E2 variant 1a (Uev1a) to catalyze the formation of Lys63-linked polyubiquitin (K63-Ub) chain, with UbcH3 to catalyze the formation of K48 polyubiquitination chain(K48-Ub), and with UbcH4, UbcH5a or UbcH5b to catalyze the formation of K11 and K48 polyubiquitin ubiquitination chains (30). Inducing the formation of K63-Ub chains to ubiquitylate IRAK1, IRAK4, myeloid differentiation factor88 (MyD88), receptor-interacting protein kinase1 (RIP1), and receptor-interacting protein kinase 2 (RIP2) (14, 22, 26, 30, 36, 37, 39, 40) demonstrate that Pellino family is a novel RING E3-ubiquitin ligase (14, 39). In addition to interacting with IRAK4, IRAK1, TAK1, TBK1, and TRAF6 (15, 32–36), each member has unique binding partners. Pellino-1, but not Pellino2 or Pellino3, has been reported to interact with MyD88 (20) and TBK1 (35). Similarly, only Pellino3 was associated with NF-κB-inducing kinase (NIK) (15, 39). Some other proteins can also interact with the Pellino family, such as Smad6/7 (20, 21), BCL10 (23), and caspase-8 (26, 27) (**Figure 1**). Upon diverse stimulation, the key biological and cellular function of the Pellino family has been identified in the innate immune system (17, 31, 41–43), namely, initiating NF-κB (44) and mitogen-activated protein kinase (MAPK) (22) to regulate the production of inflammatory cytokine and interferons (IFNs) (41), mediating cell death *via* receptor-interacting serine/threonine kinases (RIPs), and other phenotypic changes of cells and tissues (45–47).

## The Roles of the Pellino family in pattern Recognition Receptor Signaling

IL-1R, TLRs, and NLRs were involved in innate immunity to mediate the production of inflammatory cytokines (48, 49) and interferons (50). Each member of the Pellino family is crucial to PRR signaling pathways. We divided these pathways into five categories: (i) MyD88-dependent TLR/IL-1R signaling, (ii) TRIF-dependent interferons induction signaling, (iii) RIP-dependent signaling, (iv) NLR-related signaling, and (v) B-cell and T-cell signaling due to some key proteins, i.e., Myd88, TRAF6, TAK1, Toll/IL-1 receptor domain-containing adaptor inducing interferon-beta (TRIF), TBK1, RIPs, and NLRs in the signaling conduction (51).

In the *Drosophila* genome, Pellino interacts with and regulates plasma membrane MyD88-K48-Ub turnover to balance Toll-mediated immune signaling positively or negatively (52, 53). An ancestral Pellino protein from helminth species binding and poly-ubiquitinating human IRAK1 displays its E3 ligase activity and conservative function (54). In mammals, the production of proinflammatory interleukin-1 β (IL-1β), IL-6, C-X-C motif chemokine ligand 8 (CXCL8), and IFNs regulated by Pellino1, Pellino2, and Pellino3 demonstrate the key roles of the Pellino family in TLR/IL-1R signaling (25, 55–57). All of the TRIF, RIP1, RIP3, NLRs, and the Pellino family participate in the activation of NF-κB and MAPK/ERK kinase kinases (MEKKs) signaling to regulate cell survival, apoptosis, and necroptosis (4, 25, 37, 38, 57).

### Pellino Family in MyD88-Dependent TLR/IL-1R Ssignaling

TLR/IL-1R family possesses an intracellular conserved Toll/IL-1R (TIR) domain which can allow the recruitment of the adapter MyD88 for the transduction of signals (58). In this section, we mainly focus on the function of the Pellino family in MyD88-dependent TLR/IL-1R signaling.

### Pellino Family in MyD88-Dependent IL-1R Signaling

IL-1 is an important endogenous pyrogen and proinflammatory cytokine that can regulate hematopoiesis, recruit and activate neutrophils, macrophages, T and B-lymphocytes, and mediate inflammatory responses (59, 60). IL-1 induces signal conduction *via* IL-1R and IL-1R-accessory proteins to recruit MyD88-dependent signaling cascades, namely, IRAK4, IRAK1, IRAK2, TRAF6, and TAK1, that leads to the activation of the MAPKs and NF-κB (4, 61).

Pellino1, Pellino2, and Pellino3 can interact with IRAK1, TRAF6, and TAK1 (8, 32–34). Being upstream of TAK1 and downstream of IRAK1, Pellino1 is critical for the IL-1R-MyD88 dependent pathway through interaction with the IRAK4–IRAK1–TRAF6 complex (33). During this process, the catalytic activity of IRAK1 and IRAK4 is required for IL-1-stimulated activation of Pellino1 in Mouse embryonic fibroblasts (MEFs) (35). Aside from Pellino1, Pellino2 also interacted with IRAK4 (14, 57). Pellino3 physically interacts with IRAK1, TRAF6, TAK1, and NIK in HepG2 and 293 cells in an IL-1-dependent manner (15). Pellino1 and Pellino2 can replace TRAF6 to generate K63-Ub chains, activate TAK1, or induce IL-8 production *via* MyD88-IL-1β signaling in IL-1R cells that express E3 ligase-inactive TRAF6 (40).

**Figure 2:**
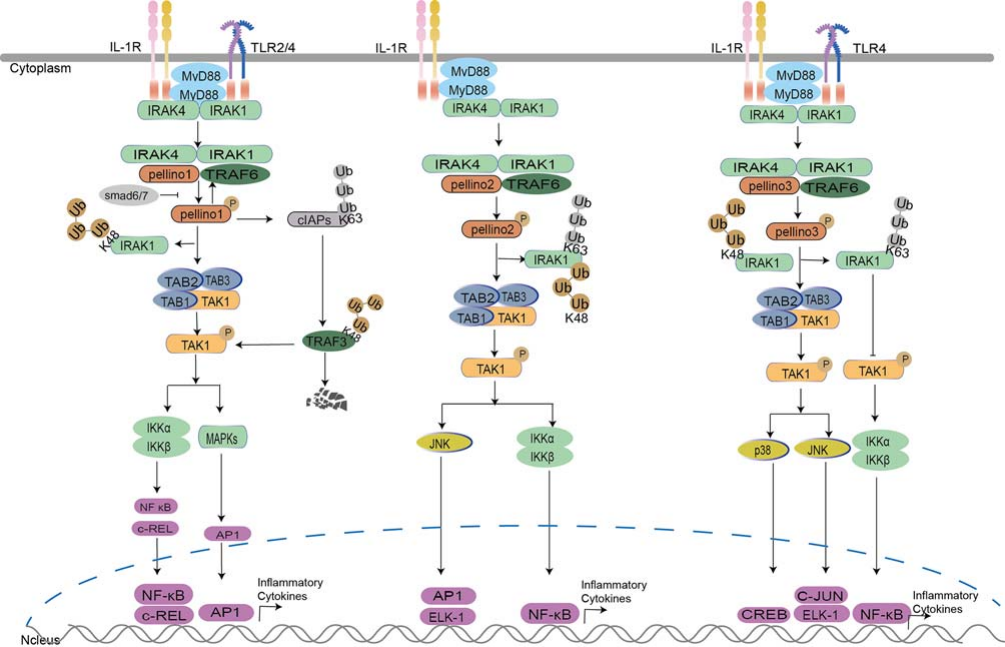
Pellino family in the Myd88-dependent TLR/IL-1R signaling. Upon IL-1 stimulation, Pellino1, Pellino2, and Pellino3 can interact with IRAK1, TRAF6, and TAK1 (8, 32–34). Pellino1 mediates the degradation of IRAK1 by K48-Ub, leading to the activation of TABs and TAK1 with the ultimate activation of NF-κB. Pellino2 leads the activation of NF-κB and JNK by successive K63-Ub and K48-Ub of IRAK1 and activation of TAK1. Pellino3 mediates the activation of JNK and p38 by K48-Ub, which leads to IRAK1 degradation and negatively regulates NF-κB activation by IRAK1 K63-Ub. In the TLR pathway, TRAF6 induces cIAPs K63-Ub to enhance TRAF3 K48-Ub degradation, elevating proinflammatory cytokine production by interrupting TRAF3 induced K48 ubiquitin-dependent degradation of c-Rel. Pellino1 can also mediate cIAPs K63-Ub to accelerate the production of proinflammatory cytokines in microglia. In macrophages, Pellino3 represses NF-κB activation by inhibiting TRAF6 downregulation. It also inhibits IRAK1 degradation *via* K63-Ub competing with K48-Ub of IRAK1, hindering NF-κB activation.

The Pellino family is associated with inflammatory mediator production (35, 62). Pellino1 knockdown can lead to a reduction in IL-1β-induced expression of proinflammatory cytokines in the bronchial epithelial cells (BEAS-2B) (62) and inhibit IL-1-mediated NF-κB activation and thus repress the production of IL-8 (33). Furthermore, *Drosophila* mothers against decapentaplegic protein 6 (Smad6) and Smad7 can bind to Pellino1 *via* mad homology (MH2) domains to mediate growth factor-β (TGF-β). It inhibited IL-1R signaling by preventing Pellino1 from forming a complex with MyD88, IRAK1, IRAK4, and TRAF6, which further suppressed IL-1β induced NF-κB activation and production of proinflammatory cytokines (20, 21). Evidence shows that Pellino1 plays a critical role in IL-1R signaling *via* MyD88, leading to NF-κB activation and proinflammatory cytokine expression. However, this conclusion is contradictory to other studies. Due to indistinct variations in NF-κB activity and expression of TNF-α, IL-6, or C-X-C motif chemokine ligand 10 (CXCL10) in mouse embryonic fibroblasts between wild type (WT) and Pellino1 knockout (KO) mice, Pellino1 is overlooked or unnecessary for the IL-1R pathway (37). A similar phenomenon can be observed in Pellino1 knockdown airway primary epithelial cells with the insignificant expression of proinflammatory cytokine CXCL8 induced by IL-1 (62). Furthermore, inactive-IRAK1induces Pellino1 significantly impaired E3 ubiquitination ligase activity with a modest effect on MAPK and NF-κB activation upon IL-1 (31). All the results indicate that Pellino1 may not be necessary for inflammation production in the MyD88 dependent IKK–NF-κB activation pathway. Whether Pellino1 is necessary for IL-1R may be controlled by cell type. Pellino2 also plays a critical role in IL-1R-mediated inflammatory production and post-transcriptional control (22), and it may be a positive regulator in the IL-1R pathway. The successive K63 and K48 ubiquitination of IRAK1 and TAK1 are required for Pellino2 to regulate IL-8 promoter activity by an NF-κB-dependent manner in the human embryonic kidney (HEK) 293-EBNA cells and the mouse embryonic fibroblast cell line C3H10T1/2 (22, 32). Upon K63 ubiquitination (22) of IRAK1 by Pellino2, the intermediate complex Pellino2–IRAK4–IRAK1–TRAF6 interacts with membrane-bound pre-associated TAK1-TGF-β activated kinase 1/MAP3K7 binding protein 1 (TAB1)-TAB2, which results in the formation of complex II (TAK1 complex, IRAK–TRAF6–TAK1–TAB1–TAB2), and IRAK1 degradation, induced by K48-linked ubiquitination of degradation. This is followed by translocation of TRAF6–TAK1–TAB1–TAB2 (complex III) from the membrane to the cytosol. TAK1 is activated and eventually leads to transcription factors activation of NF-κB, AP-1, and Elk-1in MAPKs (8, 22, 34). Pellino3 can also participate in the IL-1R signaling in HepG2 and 293 cells in an IL-1-dependent manner (15). However, Pellino3b activates JNK leading to the activation of c-Jun and Elk-1 (8, 15), and activates p38MAPK leading to cAMP-response element-binding protein (CREB) activation (24) instead of NF-κB (15). Mechanistically, upon IL-1 stimulation, upregulated Pellino3b interacts with and inhibits TAK1 complex releasing from membrane to cytosol, leading to attenuation of TAK1-dependent NF-κB activation due to Pellino3b induced K63-polyubiquitination and IL-1 induced K48 polyubiquitination competing for IRAK1-K134 ubiquitination site (8). Pellino3 activates p38MAPK *via* interacting with IRAK1, TRAF6, and TAK1. It also promotes translocation of p38 substrate MAPK-activated protein kinase (MK2) from the nucleus to the cytoplasm and activates the transcription factor CREB in a p38 MAPK-dependent manner (24). The ability of Pellino3 to activate p38 MAPK appears to be unique in the Pellino family (**Figure 2**).

For downstream signaling, Pellino1 leads to the activation of NF-κB (33) but not c-Jun N-terminal kinase (JNK) (24, 33, 34) in HEK293 cells. Mouse Pellino2 is required for NF-κB activation in mouse embryo fibroblast cells (32) and is involved in JNK signaling, which leads to AP-1 and the effect of ETS-like 1 transcription factor (Elk-1) activation in HEK293 cells (34). Pellino3 promotes c-Jun and Elk-1 activation in JNK signaling in HepG2 human hepatoma cells (15) and acts as a promoter to activate p38 MAPK in HEK293 cells (24) instead of NF-κB (15). Pellino3b, an alternative splicing variant of Pellino3, can negatively regulate IL-1-induced and TAK1-dependent NF-κB activation in synoviocytes (8) **(** **Figure 2** **)**. As a conserved E3 ubiquitin ligase family, each member activates the same or different transcription factors to regulate different downstream pathways. Perhaps each member of the Pellino family has a different division of labor upon IL-1.

### Pellino Family in MyD88-Dependent TLR Signaling

All TLRs mediate the signal conduction *via* Toll/interleukin-1 receptor (TIR) like IL-1R. Upon stimulation of ligands, several TLRs such as TLR2 and TLR4 recruit MyD88, IRAKs, TNF receptor-associated factor 3 (TRAF3), and TRAF6 to activate TAK1, leading to the activation of MAPK and NF-κB (3, 63). Upon LPS binding, TLR4 recruits MyD88, TRAF6, TRAF3, and cellular inhibitors of apoptosis proteins (cIAPs). There are two downstream signaling pathways for TRAF6. One is to activate TAK1 leading to MAPK and NF-κB activation (64–67). The other is to induce proinflammatory cytokines by stabilizing cIAPs *via* K63-Ub and then the TRAF3 K48-Ub degradation leading to the production of c-Rel (6).

An overall brain proteomes study in Pellino1 knockout mice showed that Pellino1 was involved in promoting antigen presentation, enhancing activities of adaptive and innate immune cells (68) with the contribution to microglial activation, neuroinflammatory responses, and neurological deficits through the activation of NF-κB and MAPK (42, 64, 66). Pellino1 positively regulates the production of inflammatory factors in MyD88-dependent TLR signaling (69, 70) as MyD88 deficiency hindered the expression of Pellino1, NF-κB, IL-1β, IL-6, Beclin-1, and cyclooxygenase-2 (COX-2) in a cerebral ischemia/reperfusion (I/R) mouse model (70). Pellino1 also positively regulates the MyD88-dependent pathway by promoting K63 linked polyubiquitination of IRAK1, TBK1, TRAF6, and TAK1 to active the MAPK and NF-κB signaling pathways *via* TLR2 and TLR4 pathway (69, 71). Upon LPS stimulation, the expression of Pellino1 is upregulated (69, 71, 72), possibly by increasing levels of p65 and phosphorylated IKKα/β in microglia (73). Upregulated Pellino1 activates microglia and enhances NF-κB production, MAPK phosphorylation, and proinflammatory cytokines in LPS-induced TLR4 signaling by increasing TRAF6 K63-linked ubiquitination (64–66). In addition, Pellino1 promotes K63-Ub of TRAF6 in the spinal cord to enhance morphine treatment (65). However, Pellino1 is dispensable for inflammatory responses in astrocytes (66). TRAF3 degradation contributes to the production of inflammatory factors in the MyD88-TLR pathway. Pellino1 was discovered to mediate K63 ubiquitination of cIAP, resulting in cIAP K48 ubiquitin ligase activity, ubiquitin-dependent degradation of TRAF3 (41, 74, 75) activation of microglia-mediated chemokines, and proinflammatory cytokines *via* the MyD88-dependent MAPK pathway (42, 66, 75). Pellino1 is also involved in several neurogenic diseases. Upon trans-activating protein (Tat), Pellino1 induces autophagy, interrupts expression of tight junction protein zonula occludens1 (ZO-1), and increases the permeability of the blood–brain barrier (BBB) by triggering K63-Ub of Beclin1 (76). Pellino1 also impairs microglial amyloid β-protein (Aβ) phagocytosis through promoting CCAAT enhancer-binding protein β (C-EBPβ) degradation in Alzheimer’s disease (AD) (77). In Parkinson’s disease, upregulation of Pellino1 by α-synuclein leads to the degradation of lysosomal-associated membrane protein-2 (LAMP2) and the buildup of autophagy with decreased autophagy flux by microglial exosomes (78).

Although mediated by LPS, the Pellino family plays a different role in endotoxin tolerance in macrophages. Endotoxin tolerance abrogated Pellino1 induction by LPS in macrophages (69, 71, 72) but an enhanced expression of Pellino3 (79). Elevated transcription of TNFα and IL-6 driven by TLR2/4 and also increased expression of C–C motif chemokine ligand 5 (CCL5) driven by TLR4 were observed in Pellino3-deficient human myeloid leukemia mononuclear cells (THP-1) in response to TLR agonists (79). The Pellino3 inhibits TRAF6 downregulation by reducing IRAK1 degradation *via* K63 polyubiquitination, which competes with K48 ubiquitination, resulting in NF-κB suppression (36) in J774.1 cell lines. This is consistent with prior Pellino3b results (8).

Several interesting results are discussed in the Pellino family-related MyD88-dependent TLR/IL-1R signaling (**Figure 2**). IRAK1, TRAF6, and MyD88 are crucial to the Pellino family, and the IRAK1 is responsible for the activation of the Pellino family. Pellino1 and Pellino2 can replace TRAF6 to generate K63-Ub chains. MyD88 mediates the Pellino1 expression level. Whether there is a similar phenomenon in Pellino3 needs further research. Upon the same IL-1 stimulation, the members of the Pellino family display different roles in regulating the IL-1R pathway. Pellino1 and Pellino2 are positive IKK activation regulators; however, Pellino3 is a negative regulator. This phenomenon is also present in TLR signaling upon LPS stimulation. Pellino1 significantly induced proinflammatory cytokines in microglial cells but showed no inflammatory responses in astrocytes.

Interestingly, the downregulation of Pellino1 and upregulation of Pellino3 were observed upon LPS induced endotoxin tolerance in macrophages. However, each member of the Pellino family can mediate the IKK activation or MAPK activation; not all the members act as positive roles in the signaling. Perhaps the cell type is critical in determining which member is accountable for the associated pathway, and this should be researched further.

### Pellino Family in TRIF-Dependent Interferon Induction Signaling

TRIF plays a critical role in activating NF-κB *via* a MyD88-independent pathway in TLR3 and TLR4 signaling (80, 81). In addition to NF-κB activation, TRIF can also stimulate TANK binding kinase1 (TBK1) and IKKϵ kinases to activate interferon regulatory factor (IRF) transcription factors that drive the expression of antiviral type I IFNs (80, 82). Upon LPS, poly(I:C), and viral double-stranded RNA stimulation, TRIF is recruited to promote TRAF3-dependent activation of TBK1 to activate IRF3/7 leading to the induction of IFN expression (82, 83). IRF3 and IRF7 are the most important transcription factors regulating type I IFN expression (80). Pellino1 possesses a novel function in human viral pathogen infection (41, 62, 84, 85) depending on TRIF. Pellino1, as a TLR3 positive regulator (18, 37, 86), is involved in modulating the production of proinflammatory cytokines (37, 86) and induction of IFN-I in the TLR3 pathway (41, 44, 86, 87). The deficiency of Pellino1 leads to inhibition of TLR3 and proinflammatory cytokines production but without impairing IFN antiviral induction under virus stimulation and TLR3 agonists in mice and primary bronchial epithelial cells (PBECs) (62, 86). It seems that Pellino1 is dispensable for IFN induction. However, further studies showed that Pellino1 is upregulated by TRIF, TBK1, and IKKϵ (18, 52, 69) *via* a TRIF-dependent manner in the TLR3 pathway but not the IRAKs-coupled and MyD88-dependent pathway (37, 62). Perhaps there is a priority for Pellino1 to decide which pathway to participate in. IKK ϵ and TBK1 can enhance the activation of Pellino1 depending on IRF3 (18) and K63-linked polyubiquitination of TBK1 (52, 55). As a new IRF3-dependent gene, Pellino1 enhances the interaction of IRF3 with the IFNβ promoter to promote IFN production (44). In detail, Pellino1 interacts with deformed epidermal autoregulatory factor 1 (DEAF1) independent of its E3 ligase activity, followed by DEAF1 binding to IFNβ promoters IRF3 and IRF7 to promote IFNβ gene transcription and IFNβ secretion in MEFs (88). The protein Bid can upregulate Pellino1 and enhance Pellino1 interaction with TBK1, leading to IRF3 production (73, 89).

Contrary to the above conclusion of upregulating IFNs level, Pellino1 negatively mediates the induction of IFNs in microglia *via* TRIF dependent TLRs upon the stimulation of poly(I:C), LPS, and the RNA virus in the CNS (41). Perhaps due to this, Pellino1 allows the entry and replication of West Nile Virus (WNV) in mouse macrophages, human neurons, and microglia (84), and the enhancement of ZIKA virus (ZIKV) vertical transmission and neuronal loss *in vitro* and *in vivo* (85). However, Pellino3 does not act as a mediator of proinflammatory cytokine expression in response to TLRs but as a key regulator to control TRIF dependent type I interferon expression in the TLR3 pathway by negatively regulating activation of IRF7 but not IRF3 (87). This was demonstrated in Pellino3 deficient animals, which had increased resistance to encephalomyocarditis virus and enhanced type I interferon expression but not proinflammatory cytokines in response to TLR3 activation (87). The possible mechanism is that the TLR3 induces the Pellino3 level, which interacts with and ubiquitinates TRAF6. This modification suppresses the ability of TRAF6 to interact with and activate IRF7 leading to downregulation of type I interferon expression (87). More interestingly, Pellino3 inhibits LPS-induced IFNβ expression in oxidation-low-lipoprotein (Ox-LDL) induced macrophage-derived foam cells *via* IRAK1/IRAK4/Pellino3/scavenger receptor-A1(SR-A1) dependent mono-ubiquitination of TRAF family member associated NF-κB activator (TANK) (90). In detail, Ox-LDL activates IRAK1 and Pellino3, which provokes mono-ubiquitination of the adaptor TANK in TRAF3-containing signaling complex, leading to the failure of LPS-induced TBK1 recruitment, IRF3 activation, and IFNβ expression in macrophage-derived foam cells (90).

**Figure 3:**
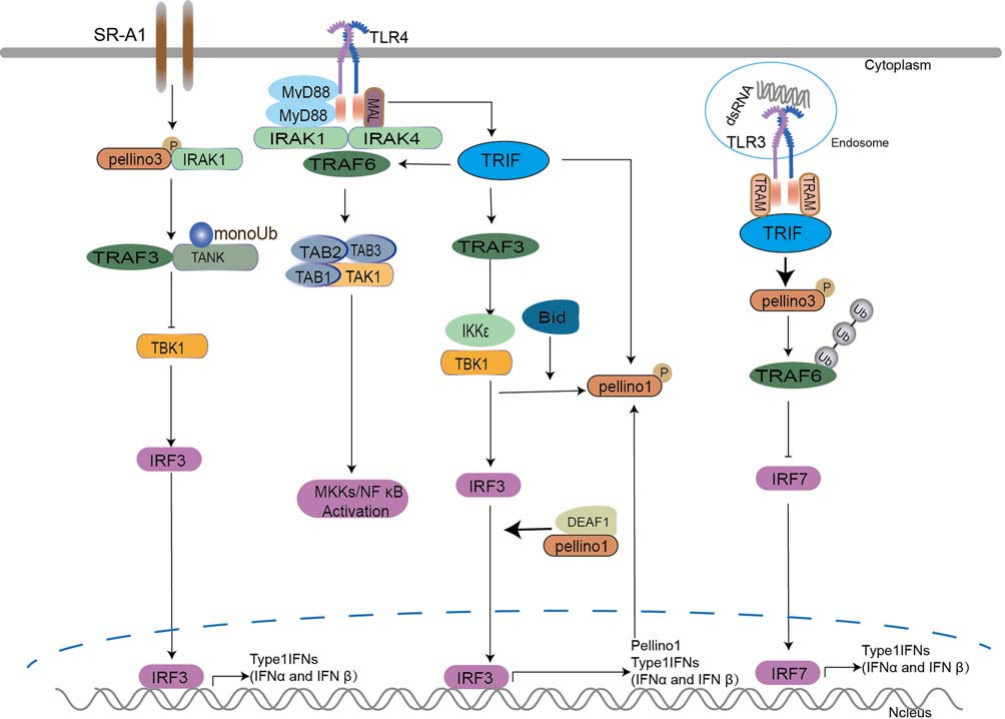
The Pellino family in TRIF-dependent interferon induction signaling. Pellino1 is required for interferon production upon viral double-stranded stimulation and is upregulated by TRIF, TBK1, and IKKϵ. IKKϵ and TBK1 enhance the activation of Pellino1 depending on IRF3 and K63 linked polyubiquitination of TBK1. Pellino1 interacts with DEAF1 independent of its E3 ligase activity and leads to the binding of DEAF1 and IFNβ promoters (IRF3 and IRF7) for IFNβ gene transcription and secretion. Bid upregulates Pellino1 and enhances the interaction of Pellino1and TBK1, leading to IRF3 production.

In the TRIF-dependent interferon induction signaling (**Figure 3**), Pellino1 and Pellion3 display polar functions in the production of IFNs (41, 44, 86, 87). The role of Pellino1 in the induction of IFNs seemed to be unclear. First, Pellino1 is dispensable or required to produce IFN in PBECs and MEFs. IFN-β induction is attenuated in myeloid cells and MEFs expressing a Pellino1 mutant lacking E3 ligase activity. Second, the fact that Pellino1 deficiency profoundly promotes IFN-β expression in microglia and Pellino1-deficient mice display heightened IFN-I levels demonstrate a potentially negative role of Pellino1 in the induction of IFN-β. The above results were confusing. The Pellino1 might play a different role in different cell types, and this needs to be further investigated. Unlike puzzled Pellino1, Pellino3 serves as a negative regulator of IRF7 but not IRF3 in TLR3 upon viruses (87). Perhaps there is a hypothesis that the Pellino family may follow a not yet clear priority rule to determine which member is responsible for the regulation of IFNs. So far, there is no report on Pellino2 and IFN production. More attention should be paid to the discrepancy of the Pellino family.

### Pellino Family in RIP-Dependent Signaling

RIP1 was initially discovered as an adapter kinase involved in the transduction of TNFR signals. It is required for the suppression of nuclear factor kappa-B kinase (IKK) activation and apoptosis *via* a RIP homology interaction motif (RHIM) in TRIF-dependent signaling (73, 91, 92) and TNF signaling in the absence of TRIF. In the TRIF-dependent pathway, RIP1 ubiquitination induced by poly(I:C) is required for IKK activation (92). The discovery of the kinase RIP3 (93, 94) and its substrate mixed lineage kinase domain-like protein (MLKL) (95, 96) leads to an awareness of this pathway. However, TRIF does not employ RIP1 to initiate IRFs (91, 92), and RIP3 is not required for NF-κB activation in TLR signaling (97). Both TNF/RIP1/RIP3/MLKL signaling and TRIF/RIP1/RIP3 pathway participate in the activation of NF-κB/MEKKs in cell survival, apoptosis, and necroptosis (4).

Pellino1 mediates RIP1 K63-Ub to active NF-κB signaling in the TRIF-dependent TLR pathway to maintain cell survival (37, 38, 98). Under LPS and dual hypoxia stimulation, destabilized Pellino1 (Ser39 phosphorylation and turnover) induced by death-associated protein kinase 1 (DAPK1) leads to the release of TRIF-RIP1 signalosome to recruit caspase-8 and induces tubular damage and cell apoptosis in acute kidney injury (AKI) model (98). The binding of Pellino1, RIP1, and TIF inhibits tubular damage by hindering cell apoptosis. In the TRIF-independent RIP pathway, IKK-related kinases activate Pellino1 in TNFα-stimulated mouse embryonic fibroblasts (MEFs) (35). According to an intriguing study, Pellino1 acts as a dual regulator of necroptosis and apoptosis. Pellino1 acts as a pro-necroptosis K63-ubiquitin ligase role in necroptosis by forming RIP1 and RIP3 complex in a RIP1 kinase activity-dependent way but as an apoptosis inhibitor by reducing expression levels of cellular FADD-like interleukin-1β converting enzyme inhibitory protein (c-FLIP) in MEFs cells stimulated by TNFα (56). In contrast to the previous result, Pellino1 might prevent HeLa cells aberrant necroptosis by causing RIP3 hyperactivation and further degradation *via* K48-linked polyubiquitylation (19). The results reflect the different roles of Pellino1 in normal and cancerous cells. However, Smad6 can block the interaction between Pellino1 and RIP1 to inhibit NF-κB (84, 85). Pellino3 is also proved to be a novel regulator of cell survival upon TNFα. Pellino3 can impair TNFα-induced complex II formation and caspase-8-mediated RIP1 cleavage *via* interacting with RIP1 and caspase 8, leading to the inhibition of apoptosis *in vitro* and *in vivo* (27).

**Figure 4:**
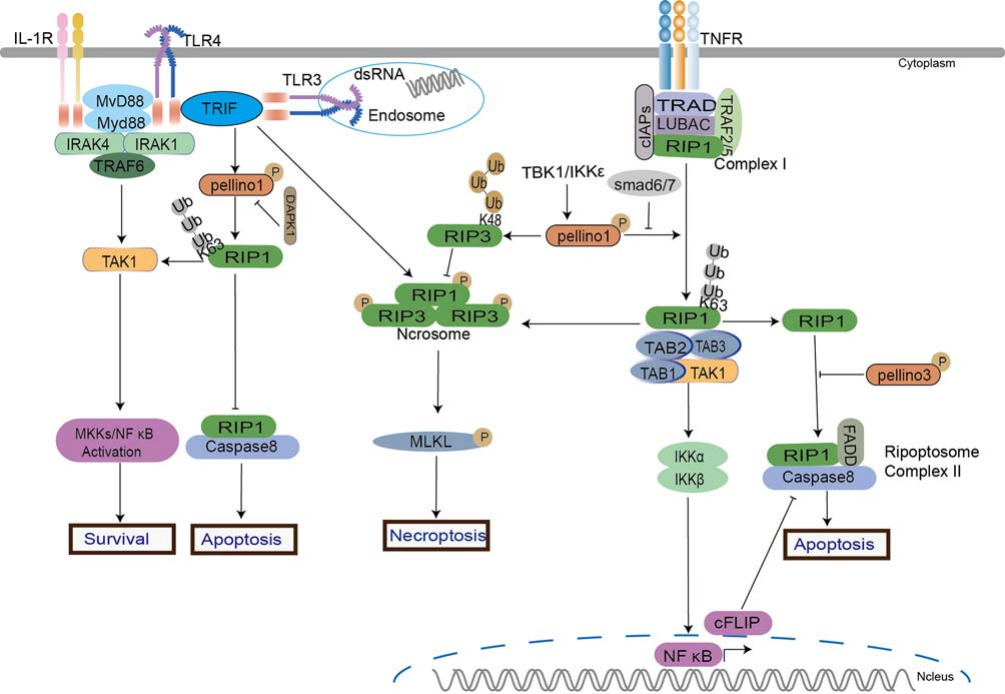
Pellino family in RIP-dependent signaling. Pellino1 induces the ubiquitination of RIP1 and RIP3 to regulate NF-κB activation in cell survival, apoptosis, and necroptosis in TNFα, TLR3, and TLR4 signaling. Pellino1 targets RIP1 by K63-Ub to active NF-κB to maintain cell survival. Under the dual stimulation of LPS and hypoxia, Pellino1 releases the TRIF-RIP1 signalosome to recruit caspase-8 and induces tubular apoptosis *via* DAPK1-mediated Pellino1 destabilization. Upon TNFα, Pellino1 is a dual modulator in necroptosis and apoptosis. Pellino1 plays a positive role in necroptosis by K63-Ub to form RIP1 and RIP3 pro-necroptosis complex in a RIP1 kinase activity-dependent way but as an apoptosis inhibitor by reducing expression levels of c-FLIP. Smad6 blocks the interaction between Pellino1 and RIP1 to inhibit NF-κB activation. Pellino1 can induce RIP3 hyperactivation and degradation *via* K48-Ub to inhibit necroptosis.

In the RIP-dependent signaling (**Figure 4**), both Pellino1 and Pellino3 were involved in two types of programmed cell death: apoptosis and necroptosis. It seemed that the K63-Ub of RIP1 is crucial to cell fate. For instance, stimulations of TLR3 and TLR4 induce the interaction of RIP1 and TRIF followed by recruitment of Pellino1, which mediates K63-linked polyubiquitylation of RIP1, leading to recruitment of TAK-1 for NF-κB induced cell survival (19, 37). Upon TNFα, the K63-linked polyubiquitylation of RIP1 is also necessary for the NF-κB pathway and cell survival (99). Pellino1 can also mediate RIP1 K63-linked polyubiquitylation on TNFα. Pellino1-induced RIP1 K63-linked polyubiquitylation appears to be a critical factor in cell survival, apoptosis, and necroptosis. Current studies show that Pellino1 only induces RIP1 K63-linked polyubiquitylation to trigger necroptosis but is not necessary for necrosome formation. Perhaps, the different interaction sites between RIP1 and Pellino1 decide the signal conduction. Interestingly, Pellion1 plays almost the exact opposite role in necroptosis in different cell types, enhancing necroptosis in normal cells (56) and preventing necroptosis in Hela cells (19). As an important role in controlling complex II formation in response to TNF, Pellino3 can interact with the complex II components, caspase-8, and RIP1, to inhibit cell death. Pellino3 plays a critical role in inhibiting the pro-apoptotic effects of TNF independent of NF-κB (27). This is consistent with previous reports indicating that Pellino3 may negatively regulate IL-1-induced and LPS-induced activation of NF-κB (8, 36). More efforts are needed to unravel the roles of the Pellino family in cell survival and death.

### Pellino Family in NLRs Related Signalings

NOD1, NOD2, and NLR Family Pyrin Domain Containing 3 (NLRP3) are involved in the anti-infection process by activating the NF-κB signaling pathway, type I IFN signaling pathway, autophagy-related pathway, and pyroptosis pathway (100, 101).

**Figure 5:**
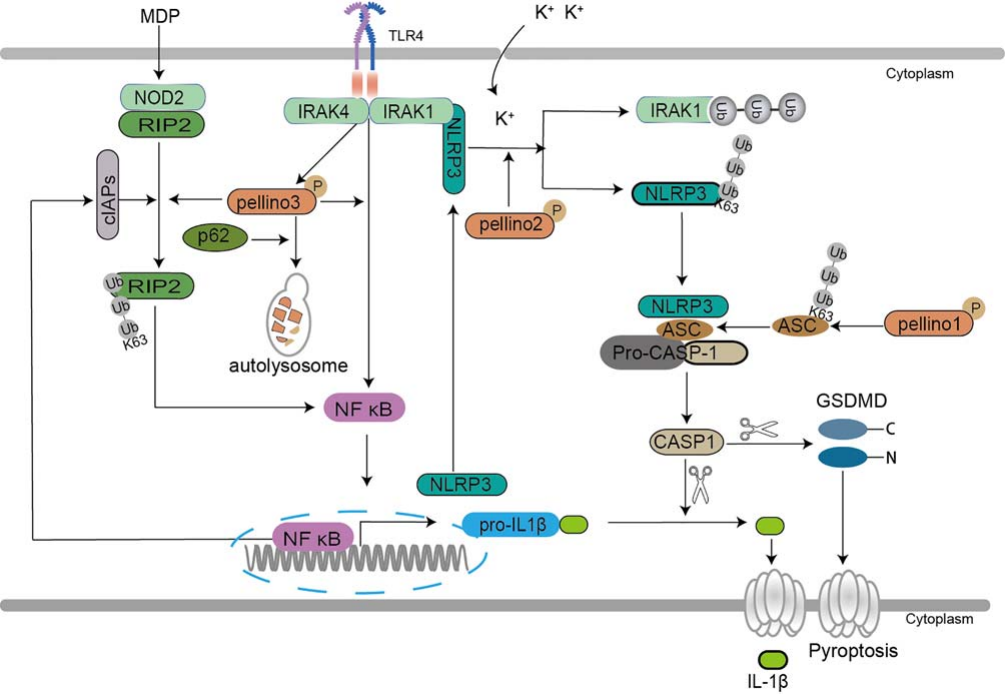
Pellino family in the NLR-dependent signaling. Pellino1 mediates the K63 ubiquitination of inflammasome adaptor ASC, which enhances the ASC/NLRP3 interaction and ASC oligomerization to facilitate NLRP3 inflammasome activation leading to induction of IL-1β secretion. Pellino2 induces IRAK1 isolation from NLRP3 *via* ubiquitination and mediates K63 ubiquitination of NLRP3 to promote the activation of NLRP3 for mature IL-1β production in response to LPS. Pellino2 can co-localize with NLRP3 and ASC during inflammasome activation in macrophages upon the effect of potassium efflux. Pellino3 acts as a potential partner of SQSTM1/p62, which leads to Pellino3 autophagy-dependent degradation in TLR4-signaling, thereby impairing Pellino3-dependent pro-IL-1B proinflammatory expression. Pellino3 promotes MDP-induced K63 ubiquitination of RIP2 and recruits TAK1 and IKK complexes to active NF-κB and MAPK in an IAP-independent manner to maintain cell survival.

In NOD2 related signaling, Pellino3 exerts a protective function *via* NOD2 in chemical drugs induced models of colitis (26). Pellino3 promotes magnesium-dependent phosphatase (MDP) induced K63-Ub of RIP2 and recruits TAK1 and IKK complexes to active NF-κB and MAPK in an inhibitor of apoptosis family of protein (IAP)-independent manner to maintain cell survival (26) (**Figure 5**).

Two pathways are involved in IL-1β secretion, TLR/IL-1R-mediated upregulation of precursor pro-IL-1β and NLR-induced activation of caspase-1 that cleaves pro-IL-1β to yield mature IL-1β secretion (102). Pyroptosis is a novel programmed cell death featured by IL-1β secretion (103). In NLRP3-related pyroptosis, the oligomerization of NLRP3, pro-caspase-1, and the inflammasome adaptor apoptosis-associated speck-like protein containing a caspase recruitment domain (ASC) causes pro-caspase-1 to be converted into active caspase-1, which then cleaves pro-IL-1β and pro-IL-18, resulting in the maturation and secretion of proinflammatory cytokines (104). Pellino1, Pellino2, and Pellino3 are demonstrated to mediate the release of IL-1β and IL-18 in cell pyroptosis (57, 105, 106). A new study reveals that Pellino1 is required for NLRP3-induced caspase-1 activation and IL-1β maturation (106). Pellino1 increases NLRP3 inflammasome activation, which results in IL-1β production, by facilitating ubiquitination of the inflammasome adaptor ASC K63, enhancing the ASC/NLRP3 interaction and ASC oligomerization (106). Pellino2 is also essential for the priming and activation of inflammasome to induce pyroptosis (57, 105). In Pellino2 deficient macrophages, the activation of the NLRP3 inflammasome is suppressed (57). Pellino2 isolates IRAK1 from NLRP3 *via* ubiquitination and mediates K63 ubiquitination of NLRP3 to increase NLRP3 activation for mature IL-1β generation in mice and bone marrow-derived macrophages (MDMs) in response to LPS (57). Further studies show that Pellino2 can co-localize with NLRP3 and ASC during inflammasome activation in macrophages upon the effect of potassium efflux (105). Both Pellino1 and Pellino2 are implicated in NLRP3-mediated pyroptosis, demonstrating the importance of the Pellino family in pyroptosis. Furthermore, the autophagy-dependent degradation of Pellino3 induced by sequestosome-1 (SQSTM1/p62) hindering IL-1β secretion upon LPS offers a strong backup for the roles of the Pellino family in pyroptosis (25).

The above results show that the Pellino family is crucial to ubiquitin-dependent inflammasome activation and inflammatory release (**Figure 5**). Previous studies reported that the Pellino family is a key mediator for activation of NF-κB (37), and NF-κB is involved in NLRP3 induction. The most surprising is that Pellino1 deficiency did not reduce the induction of NLRP3 expression (106). Pellino1, Pellino2, and Pellino3 may play a role in the division and cooperation to mediate NF-κB activation, inflammasome activation, and inflammatory release in pyroptosis. In NOD2 related signaling, Pellino3 is still a protective regulator to maintain cell survival, consistent with Pellino3 in TNF signaling. Perhaps Pellino3 may differ from Pellino1 and Pellino1 in special cells and contexts. More efforts are needed to reveal the roles of the Pellino family in programmed cell death.

### Pellino Family in B-Cell and T-Cell Signaling

**Figure 6:**
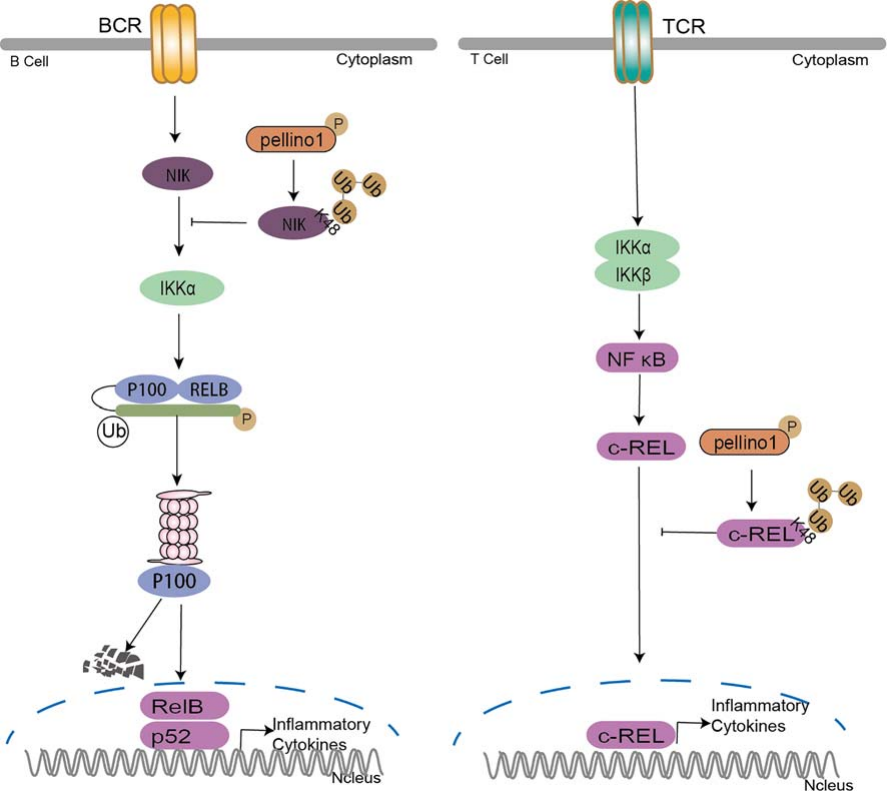
Pellino family in B cell and T cell. Pellino1 inhibits noncanonical NF-κB activation by K48 ubiquitination of NIK to downregulate nuclear p52 and Rel B in the noncanonical NF-κB pathway. Pellino1 also negatively regulates T cell activation and prevents autoimmunity by specific K48 ubiquitination of c-Rel to inhibit NF-κB activation.

In addition to the above functions in immunity, Pellino1 shows a potent negative function in T cell and B cell activation (43, 107). Under normal circumstances, Pellino1 is highly expressed in mouse splenic B cells and T cells (107). Pellino1 inhibits T cell activation and prevents autoimmunity by ubiquitinating c-Rel, a downstream important protein in NF-κB activation, with specific K48-Ub (107). Pellino1 is seemed to be unique for T cell activation and maintenance of peripheral immune tolerance for its high expression in lymphocytes (107). Pellino1 deficiency promoting B cell activation hints that Pellino1 negatively regulates B cells specifically in response to poly(I:C) and noncanonical NF-κB stimulation (43, 108). Pellino1 inhibits noncanonical NF-κB activation and alleviates lupus-like disease in systemic lupus erythematosus by K48 ubiquitination of NIK to downregulate nuclear p52 and Rel B (43) (**Figure 6**).

Ubiquitination has emerged as a critical mechanism regulating T cell and B cell activation (109). Pellino1 is critical in regulating IKK activation by TRIF dependent TLR signaling, although it is largely dispensable for IKK activation in MyD88-dependent TLR/IL-1R (37). However, in B-cell and T-cell **(** **Figure 6** **),** the reason it is dispensable to active IKK by TCR signals may be the degradation of c-Rel induced by Pellino1specific K48 ubiquitination (107). The noncanonical NF-κB pathway critically regulates B cell activation and antibody production. It is reported that TRAF2-cIAPs mediated the K48 ubiquitination of NIK as E3 ligases (110–112). Pellino1 is also required for TLR-induced cIAPs ubiquitination and activation in microglia (75). So it is reasonable to assume that Pellino1-mediated NIK ubiquitination may be due to the activation of cIAPs by Pellino1.

## Pellino Family in Tumor and MicroRNAs Related Signalings

### Pellino Family in Tumorigenesis

Pellino1 plays a novel role in angiogenesis, a typical phenotype in tumorigenesis (113).

As a downstream of vascular endothelial growth factor receptor 2 (VEGFR2), Pellino1 induces the AKT and MAPK activated protein kinase 2 (MK2) phosphorylation to restore cell migration potential, proangiogenic responses and the wound healing ability with VEGFR2 deficiency *in vitro* and *in vivo* (114). Further studies demonstrated that Pellino1 is a novel proangiogenic molecule directly regulated by VEGFR (115). In mouse ischemia models, Pellino1 deletion increases oxidative stress, reduces cIAP2-NF-κB cell survival, decreases angiogenic response, and lowers tissue function (116). Transgenic mice constitutively expressing human Pellino1 had a shorter lifespan, a wide range of lymphoid tumors, and prominent B-cell infiltration (117). Pellino-1 may be an oncogene in cancer based on its proangiogenic and tumor development function. The association of Pellino1 with protooncogene-MYC, B cell lymphoma 6 protein (BCL6), murine double minute X (MDMX), and p53 demonstrates the role of Pellino1 in cancer (117–120). In diffuse large B-cell lymphoma, Pellino1 directly interacts with and induces oncoprotein BCL6 K63-Ub (117). Pellino1 is required for DNA damage in the promotion of HR repair by feedback activation of ataxia telangiectasia-mutated gene (ATM) *via* NBS1 ubiquitination (121) and p53 activation upon exposure to DNA damaging agents (120). Pellino1 negatively regulates and sequesters MDMX *via* ubiquitylation in the cytoplasm and free p53 to activate responsive genes such as p21 (119). Furthermore, Pellino1 downregulation causes MDMX nuclear localization, lowers p53 activity, and speeds up c-MYC-induced carcinogenesis linked with a reduction in p53 function (119). IAP may be a positive partner of Pellino1 in regulating tumor cell survival (116, 122). High expression of Pellino1 in human lung cancer cell lines upregulates the expression of IAP proteins (cIAP1 and cIAP2) through K63-Ub, which leads to cell survival but not apoptosis (122). Pellino1 can also promote epithelial-mesenchymal transition (EMT) by inducing K63-Ub of Snail and Slug, contributing to tumorigenesis (47, 123). Fundamentally, Pellino1 causes homeostatic regulation of the mitotic cell cycle and checkpoints to be inhibited, contributing to the initiation and progression of the neoplastic chromosome aneuploidy through ubiquitination-mediated downregulation budding uninhibited by benzimidazoles related1 (BubR1) and induced mitotic dysfunction (124). This may be crucial evidence to demonstrate Pellino1 to be an oncogene.

As a positive regulator of inflammatory factors, Pellino1 induces the production of inflammatory factors followed by the change of inflammatory microenvironment leading to the transformation of normal cells to tumor cells. So it is necessary to study the inflammatory microenvironment induced by the Pellino family in normal cells, tumor cells, and even cell co-culture systems.

### Pellino Family in microRNAs Related Signalings

MicroRNAs (microRNAs) are small non-coding RNAs with the capability of modulating gene expression at the post-transcriptional level either by inhibiting messenger RNA (mRNA) translation or by promoting mRNA degradation (125). Several microRNAs are involved in interactions with the Pellino family. MicroRNA-21 (126–128), microRNA-153-3p (129), and microRNA-155 (130, 131) are involved in T cell regulation; MicroRNA-590-5p (132), microRNA-142a-3p (133), microRNA-155-5p (133), and microRNA-744 (134) in inflammatory disease; MicroRNA-128-3p (134) in tumor disease.

A positive correlation between microRNA-21 and Pellino1 suggests that microRNA-21 and Pellino1 might be associated with autoimmune primary ovarian insufficiency (POI) patients (126). MicroRNA-21 targets the Pellino1–c-Rel pathway to promote glucose metabolism of pathogenic T helper cell 17 (TH17) cells by activating the NF-κ B with a decrease in Pellino1 and an increase in c-Rel (128). In systemic lupus erythematosus, upregulated microRNA-153-3p represses Pellino1 *in vitro*. It induces immune dysregulation by lowering umbilical cord mesenchymal stem cells (UC-MSCs) proliferation, migration, and mitigates the decrease in T follicular helper (Tfh) cells and increases T regulatory (Treg) cells (SLE) (129). MicroRNA-155 represses the expression of Pellino1, leading to the abrogation of the c-Rel, which controls cellular proliferation and CD40L expression in Tfh cells (130). MicroRNA-155 (131), microRNA-590-5p (132), microRNA-142a-3p (133), and microRNA-155-5p (133) can all target and reduce Pellino1 expression, leading to the suppression of pro-inflammatory production in neuroinflammation. MiR-744 interacting with the 3’ untranslated region (UTR) represses Pellino3 expression and leads to upregulation of the IFN-dependent chemokines C–C Motif Chemokine Ligand 5 (CCL5) and CXCL10 (135). In non-small cell lung cancer, levels of Pellino3 are positive to the long non-coding RNA (lncRNA) MIAT but negatively related to miR-128-3p (134). It is clear that microRNAs primarily suppress the expression of Pellino1 to modulate immune responses. More research should be conducted to determine the association between microRNAs and the Pellino family.

## Conclusion

As a highly conserved protein and positive regulator in immunity discovered in *Drosophila* (52), the structure of Pellino in other species is also conserved, e.g., viral Pellino (136), Freshwater Prawn (137), Zebrafish (138), *Crassostrea hongkongensis* (139), and *Japonicas* (140). Viral Pellino should be studied further for a poxviral homolog of the Pellino protein capable of inhibiting Toll-like receptor signaling independent of IRAK1 and inhibiting Pellino3-mediated activation of the p38 MAPK pathway (136). The function of viral Pellino suggests that the mammalian Pellino family may act as a barrier or enhancer during viral infection.

There are two important conserved domains for the Pellino family: the FHA domain promoting phosphorylation with IRAKs (16, 24, 28–31)and the RING domain, which determines E3 ligase features (7, 14, 39). The FHA domain in the Pellino family differs from the classical FHA domain by containing an additional appendage or “wing” that is formed by two inserts in the FHA region (16). Interestingly, multiple IRAK phosphorylation sites in the “wing” region and the importance of this appendage region for IRAK binding remain to be experimentally addressed. More interesting is that different domains can interact with the same protein. Pellino1 can interact with RIP3 depending on the FHA and RING-like domains (19, 37). The FHA and RING-like domains are responsible for Pellino3 interacting with RIP1, RIP2, and caspase-8 (26, 27). These suggest that the activation of different sites may be a key factor in determining the cell to survive or be dead dependent or independent on the RIP family.

There are some conflict points about the Pellino family in regulating PRR signalings. In contrast to the positive role in regulating proinflammatory cytokine induction (37), Pellino1 negatively regulates T-cell activation in autoimmunity (107); and promotes microglia-mediated CNS inflammation (75) by negatively regulating type I interferon induction and antiviral immunity in the microglial cells (41). Pellino1 and Pellion3 display polar functions in the induction of IFNs (41, 44, 86, 87). Unlike peripheral macrophages expressing Pellino1, Pellino2, and Pellino3, microglia predominantly express Pellino1 (75). The induction of IFNs in MEFs and peripheral cells induced by Pellino1 deficiency showed no significance (37). However, IFN-β induction is attenuated in myeloid cells and MEFs expressing a Pellino1 mutant lacking E3 ligase activity (44). The more intriguing aspect is that Pellino1 performs different roles in necroptosis and apoptosis in the same cell, as a critical modulator of TNF-α-mediated cell death pathways, enhancing necroptosis and inhibiting apoptosis by modifying K63-Ub of RIPK1 with the inconstant expression of c-MYC and c-FLIP (56). These indicate that specific tissue expression of Pellino1 may promote their specialized roles in specific cells. According to the current studies, the Pelino1 tissue expression level is from high to low in peripheral blood, leukocytes, placenta, lung, liver, kidney, spleen, thymus, skeletal muscle, brain, small intestine, colon, and heart (33). Pelino3 tissue expression level is from high to low is brain, testis, heart, liver, lung, placenta, stomach, kidney, spleen, small intestine, colon, and muscle (15). Perhaps the tissue expression levels of Pellino1 and Pellino3 may be a clue to explain the polarized function of the Pellino family. More attention should be paid to Pellino2 and Pellino3 for a better understanding of the roles of the Pellino family.

Several studies have shown that Pellino1 acts as an oncogene role in tumorigenesis to maintain cell survival (116, 122) and even upregulates other oncogene levels, e.g., Bcl6 and c-Myc (122). As a positive regulator of inflammatory factors, Pellino1 induces the production of inflammatory factors followed by the change of inflammatory microenvironment leading to the transformation of normal cells to tumor cells. It is necessary to study the inflammatory microenvironment induced by the Pellino family in normal cells, tumor cells, and even cell co-culture systems.

Only a few proteins have been reported to mediate the Pellino1, e.g., Smad6/7 (20, 21), DEAF1 (88), Bid (89), and DAPK1 (98), which positively or negatively regulate the Pellino family. A new study reports six novel interaction partners of Pellino-2 in the liver cells, insulin receptor substrate 1 (IRS-1), NIMA-related kinase 9 (NEK9), tumor necrosis factor receptor-associated factor 7 (TRAF7), roundabout homolog 1 (ROBO-1), and disheveled homolog 2 (DVL-2) (141). More efforts are needed to study the expression and binding partners of the Pellino family in both the immune cells and non-immune cells. Understanding the regulatory mechanism between the Pellino family and other proteins can assist us in acquiring a comprehensive knowledge of the cross-talk among PRRs signaling.

This paper mainly reviews the roles of the Pellino family in the PRR signaling pathways. According to a flow of studies, we can preliminarily infer that the Pellino family has indeed been involved in the PRRs related pathways with the major function of regulating IFNs and inflammatory factors leading to cell survival or death. Maybe the different cell types and ligands stimulation play crucial roles in the Pellino family-related PRRs signalings. However, there are still many contradictory phenomena that cannot be explained very well. The Pellino family might play different roles in different cell types and contexts. Currently, Pellino1 has attracted a lot of attention and more efforts will be needed to study Pellino2 and Pellino3 in order to have a better understanding of the whole family in immunity. The future focus is to probe a more detailed and clear mechanism of the Pellino family in the immune system to improve related immune diseases.

## Author Contributions

EZ contributed ideas, writing the initial manuscript and creating the figures. XL revised the manuscript, approved the final version and received research support. All authors listed have made a substantial, direct, and intellectual contribution to the work and approved it for publication.

## Funding

This work was supported by grants from the National Natural Science Foundation of China (No. 82073870) and the Shandong Provincial Natural Science Foundation (No. ZR2019MH001).

## Conflict of Interest

The authors declare that the research was conducted in the absence of any commercial or financial relationships that could be construed as a potential conflict of interest.

## Publisher’s Note

All claims expressed in this article are solely those of the authors and do not necessarily represent those of their affiliated organizations, or those of the publisher, the editors and the reviewers. Any product that may be evaluated in this article, or claim that may be made by its manufacturer, is not guaranteed or endorsed by the publisher.
